# Impending hand compartment syndrome in an infant: is clinical evaluation enough to perform a fasciotomy?

**DOI:** 10.1002/ccr3.2644

**Published:** 2020-01-22

**Authors:** Ioannis Delniotis, Christos Gekas, Benedikt Leidinger, Alexandros Delniotis, Nikiforos Galanis

**Affiliations:** ^1^ Department of Paediatric Orthopaedics Orthopaedic Clinic Volmarstein Wetter (Ruhr) Germany; ^2^ Department of Orthpaedics and Traumatology Papageorgiou General Hospital Thessaloniki Greece; ^3^ Aristotle University of Thessaloniki AUTH Thessaloniki Greece; ^4^ Department of Orthpaedics and Traumatology Hippokration General Hospital Thessaloniki Greece; ^5^ Department of Physical Therapy Alexander University of Thessaloniki Thessaloniki Greece

**Keywords:** compartment syndrome, early diagnosis, hand, infant

## Abstract

The purpose of this case report is to raise awareness of the early diagnosis and treatment of compartment syndrome in children. Late diagnosis can lead to irreversible outcomes, including myonecrosis, neurologic injury, functional problems, and even amputation. In this age group, clinical judgment may be enough to proceed with fasciotomy.

## Graphical abstract

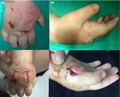

## 1 CAN I RELY ON MY CLINICAL JUDGEMENT TO DIAGNOSE A COMPARTMENT SYNDROME IN CHILDREN?

**Figure 1:**
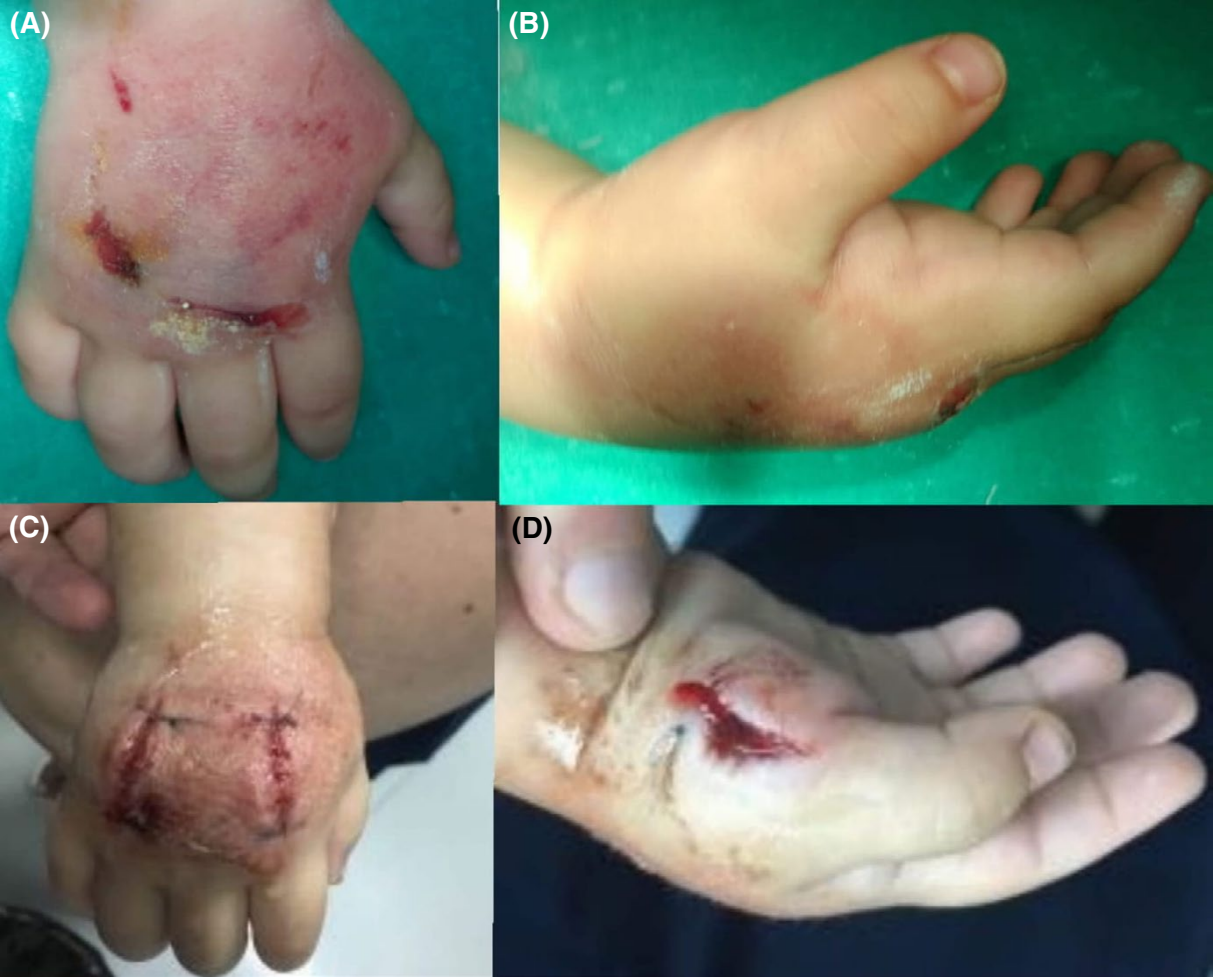
A‐D, 1A and 1B show the clinical presentation of our patient with impending hand compartment syndrome. Severe swelling and ecchymosis are obvious. Figure 1C and 1D show the dorsal incisions (in line with 2nd and 4th metacarpals) and an incision along the radial side of the 1st metacarpal (to release the thenar compartment). Additional incisions (hypothenar compartment and carpal tunnel release) may be needed

A 12‐months‐old infant (male) presented to our department, with progressive swelling and pain on his right hand, after trauma. The patient's hand was accidentally caught between an elevator door and the wall. Initial examination (1.5 hours after injury) revealed moderate to severe swelling and ecchymosis of the hand (Figure 1A and 1B). The patient was capable of performing flexion of the fingers, but the range of motion was limited. Sensation and neurovascular examination were intact except for a prolonged capillary refill (>2 seconds). Due to the age of our patient and the pain, the clinical examination was extremely difficult to draw safe assumptions. X‐rays were taken, but no fracture was obvious.

The above case scenario describes an impending compartment syndrome of the hand in an infant, which is an extremely rare clinical situation. Compartment syndrome means high pressure within muscle compartments that impairs perfusion, and the most common reasons in children are fractures, crush injuries, burns, infection, tight casting, and intravenous infiltrations (iatrogenic nature).^1^ In children, the actual amount of pain cannot be estimated and an accurate clinical examination is difficult, if not impossible. The well‐known 5 “P’s” (pain, pallor, paresthesia, paralysis, and pulselessness) are unreliable signs in children. Increasing analgesic needs is a more sensitive indicator of an impending compartment syndrome.^2^

A high index of suspicion, increasing pain requirements and increasing swelling, can be the only clinical findings that a doctor can rely on, to diagnose a compartment syndrome in children, perform fasciotomy (Figure 1C and 1D), and avoid disastrous outcomes. Regarding our patient, at 6‐month follow‐up neurological and vascular examination was normal.

## INFORMED CONSENT

Informed consent was obtained from the patient's parents for the photographs to be taken and published in this article, without identifying information.

## CONFLICT OF INTEREST

There are no conflicts of interest associated with this publication, and there has been no financial support for this work that could have influenced its outcome.

## AUTHOR CONTRIBUTIONS

ID: drafted the manuscript and contributed to patient care. CG: obtained the photographs and contributed to patient care. BL: revised the manuscript. AD: drafted the manuscript. NG: final approval of the version to be published.
